# The Combination of Shading and Potassium Application Regulated the Bulb Active Ingredients Accumulation in *Fritillaria thunbergii* Miq. by Affecting Rhizosphere Microecology

**DOI:** 10.3390/microorganisms13010125

**Published:** 2025-01-09

**Authors:** Leran Wang, Bingbing Liang, Jia Liu, Huizhen Jin, Zixuan Zhu, Siyu Hao, Shumin Wang, Xiaoxiao Sheng, Xinshu Zhou, Honghai Zhu, Ning Sui

**Affiliations:** 1 School of Pharmaceutical Sciences, Zhejiang Chinese Medical University, Hangzhou 310053, China; wanglrnjau@163.com (L.W.); liangbingbing0510@163.com (B.L.); 15205178390@163.com (J.L.); jhz99804@163.com (H.J.); zzx19861403474@163.com (Z.Z.); haosiyu1006@163.com (S.H.); wsm000105@163.com (S.W.); shengxiaoxiao2023@163.com (X.S.); 15706816756@163.com (X.Z.); 2 School of Life Sciences, Zhejiang Chinese Medical University, Hangzhou 310053, China; 3 Jinhua Academy, Zhejiang Chinese Medical University, Jinhua 321000, China

**Keywords:** *Fritillaria thunbergii* Miq., rhizosphere microorganisms, active ingredients, shade, potassium application

## Abstract

The bulbs of the lily plant *Fritillaria thunbergii* Miq. possess substantial medicinal properties for relieving coughs and clearing the lungs. However, excessive pursuit of yield during cultivation has led to a decrease in medicinal ingredients. Therefore, we aimed to investigate the effects of two single-factor treatments, shading (SK0) and potassium application (S0K), and their coupling treatment (SK) on bulb biomass and medicinal substance content, along with the role of rhizosphere microorganisms. Shading increased the content of active ingredients in bulbs by approximately 11.7% while decreasing bulb biomass by approximately 11.3%. SK treatment mitigated the biomass reduction caused by SK0 treatment while enhancing the accumulation of active ingredients in *F. thunbergii*, up to 1.2 times higher than that of SK0 treatment. In rhizosphere soil, *Allorhizobium–Neorhizobium–Pararhizobium–Rhizobium* (ANPR), *Chryseobacterium*, *Brevundimonas*, and *Phoma* exhibited significant positive correlations with medicinal components, among which ANPR, *Brevundimonas, Chryseobacterium,* and *Phoma* were responsive to SK treatments. Also, *Burkholderia–Caballeronia–Paraburkholderia* (BCP) and *Brevundimonas* responded to changes at different growth stages of *F. thunbergii.* The relative abundance of these microorganisms was associated with the alterations of soil factors resulting from shading or K application. Our results indicate that these microorganisms are beneficial to the growth of bulbs and the synthesis of active components in *F. thunbergii.* The combination of shading and K application may regulate the accumulation of medicinal substances in *F. thunbergii* by modulating the structure of the soil microbial community. Our results serve as a reference for soil improvement for medicinal plant cultivation.

## 1. Introduction

*Fritillaria thunbergii* Miq. belongs to the lily family, and its bulbs have been used in traditional Chinese medicine for over 2000 years to relieve coughs and clear the lungs [1]. Modern pharmacological studies have proved that *F. thunbergii* has antitussive, expectorant, anti-inflammatory, antioxidant, and anticancer properties [2,3]. Importantly, it is effective in treating COVID-19 pneumonia [4]. *F. thunbergii* is also valued in Asian countries such as South Korea and Japan because of its medicinal value and horticultural use [5,6]. In recent years, the quality and yield of the *F. thunbergii* planting industry has been unstable, strongly restricting its sustainable development of the *F. thunbergii* industry [7]. Therefore, improving quality and stabilizing yield are crucial issues that must to be addressed in *F. thunbergii* production.

Wild *F. thunbergii* resources are primarily found in forested areas or on the shaded slopes of hills, and are characterized by high quality but low yield. Owing to the limited availability of wild resources, all *F. thunbergii* on the market are cultivated varieties. Shading can be used to simulate the natural survival conditions of medicinal plants. Various studies on medicinal plant cultivation have reported that shading enhances the quality of medicinal plants. Moderate shading can increase the content of active ingredient in medicinal plants, such as *Angelica dahurica* [8], *Bletilla ochracea* [9], and *Scutellaria lateriflora* [10]. However, in crop shading experiments, reduced yields have been observed, including decreased biomass following colored rice shading [11]. There have been substantial reductions in the dry matter quality and yield of wheat, affecting plant growth and development [12], with similar findings in medicinal plant experiments [13]. Therefore, although shading can improve the quality of medicinal plants, it is important to address its adverse impact on biomass and yield.

Potassium (K) is a crucial macronutrient for crop growth. Applying K fertilizer improves root growth, carbohydrate synthesis, transportation, storage, and overall crop quality [14]. Appropriate topdressing of K fertilizer can promote the growth and thickening of the main ginseng root, and increase ginseng root weight per plant and yield [15]. The yield of *Scutellaria baicalensis* increases with higher doses of K fertilizer, leading to an increase in scutellarin content [16]. Previous studies in this laboratory have shown that proper application of K fertilizer can help improve both the yield and effective medicinal components’ content of *F. thunbergii* [17]. Therefore, increasing K fertilizer use may be beneficial in addressing issues related to yield decline caused by shading.

To date, studies on shading and K application to plants have mainly focused on the effects on plant aboveground biomass and yield, with relatively little focus on changes in the rhizosphere microenvironment of underground parts. Given that the medicinal parts of some plants, such as *F. thunbergii*, are located underground, it is important to investigate the growth and quality of *F. thunbergii* from the perspective of its rhizosphere microenvironment. The rhizosphere is the junction between the plant and the soil. Rhizosphere microorganisms can promote plants’ growth by absorbing nutrients from the soil. It produces plant hormones through its own metabolism to promote plant growth. They can participate in regulating the expression of active substance synthesis genes or related enzymes in plants, which affects their secondary metabolism [18,19,20,21]. Fertilization treatments have been studied extensively for plant rhizosphere microorganisms. Applying K and N fertilizers can increase the number of bacteria in the soil and soil enzymes, which can improve soil fertility and promote the growth of medicinal plants, yield formation, and effective component content [22,23]. Light also influences on soil physical and chemical properties and soil enzymes, and it may also indirectly affect the rhizosphere microenvironment of plants. Therefore, it is necessary to study the impact of shading, K application, and their combination on the rhizosphere microorganisms of *F. thunbergii*.

In this study, we established a coupling experiment with the aim of investigating the effects of shading and K application on *F. thunbergii* yield and quality from the perspective of rhizosphere microorganisms. The purpose of this study was to address the following research questions: whether shading, K application, and their combination can improve the growth, development, and quality of *F. thunbergii*; how shading, K application, and their combination affect the rhizosphere microecology of *F. thunbergii*; whether shading, K application, and both combined influence the growth, development, and medicinal components of *F. thunbergii* through rhizosphere microorganisms. It also aimed to identify specific key microorganisms and their dynamic changes at different stages. The findings provide theoretical and technical support for promoting high quality, stable yield, ecological cultivation, and regulation of *F. thunbergii*.

## 2. Materials and Methods

### 2.1. Experimental Design and Materials

**Figure 1:**
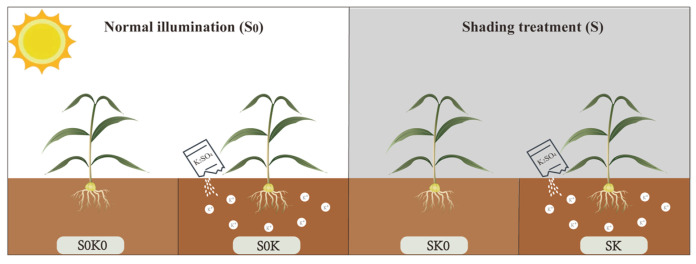
Schematic diagram of the experimental treatments.

The experiment was conducted from September 2022 to May 2023 in Shaoxing, Zhejiang, China (29.60° N, 120.35° E). The experiment used *F. thunbergii* as the experimental material. This experimental material source information is in the Taxonomy data- 104 base, and the Taxonomy ID number is 108546. The field experiment was conducted using a split-plot design and replicated three times. The main plots were shading treatments (shading 0% and shading 50%), and subplots contained two topdressing K application levels (0 and 22.5 kg K_2_O ha^−1^). The potassium fertilizer selected is potassium sulfate, which was applied on the 15th day and 40th day after the emergence of seedlings. Shade treatment was conducted during the first application of potassium fertilizer, with a total of four treatments and three replicates for each treatment. There were four treatments (Figure 1) in this experiment, which were shading 0% and topdressing potassium 0 kg K_2_O ha^−1^ (S0K0), shading 0% and topdressing potassium 22.5 kg K_2_O ha^−1^ (S0K), shading 50% and topdressing potassium 0 kg K_2_O ha^−1^ (SK0), shading 50% and topdressing potassium 22.5 kg K_2_O ha^−1^ (SK), each with three replicates. In this experiment, we used the bulbs and rhizosphere soil of *F. thunbergii* from the pre-treatment period (PT, seedling stage) and the two post-treatment periods (T1, flowering stage; T2, bulb expansion stage) as the samples, and 15 holes were randomly selected for each treatment group. Each primary plot measured 10 m × 3 m, with a row spacing of 0.15 m and an interplant spacing within the row also set at 0.15 m. Nitrogen fertilizer, phosphorus fertilizer, and potassium fertilizer, and organic fertilizer were used as basal fertilizer in all treatments, and the application rates were applied according to the actual production and remained consistent.

### 2.2. Sample Collection and Processing

The rhizosphere soil collection method described by Beckers [24] was adopted. The soil samples in the root zone of *F. thunbergii* were dug out with a shovel, and the soil outside the root system was shaken off. The root system of *F. thunbergii* was placed in a 50 mL sterile centrifuge tube containing 30 mL PBS solution, and the ultrasound was performed for 2–3 min. The root system in the 50 mL centrifuge tube was picked out using sterile tweezers, and the remaining suspension was centrifuged at high speed for 20 min. The rhizosphere soil was collected, frozen in liquid nitrogen, and stored in a refrigerator at −80 °C for the determination of the microbiome. The soil samples collected near the root surface were placed in a sealed bag with a brush and air-dried in a cool location. After drying, the soil was sieved through a 1 mm sieve and divided into two parts. One part was stored in a 4 °C refrigerator for soil enzyme activity determination, while the other part was sealed and stored in a cool place for soil factor determination. The cleaned bulbs were placed in envelopes and heat-treated at 60 °C for 30 min, followed by drying at 55 °C for 2 days to maintain the sample water content below 12.0%, and then weighed to obtain the biomass.

### 2.3. Determination of Medicinal Active Ingredients

The air-dried medicinal materials were crushed into powder samples passing through the No. 4 Pharmacopoeia Sieve. According to the method of Ning Sui [17], 2 g of dried powder of *F. thunbergii* was weighed and placed in a flask, and 4 mL of concentrated ammonia test solution was added for infiltration for 1 h. Then, 40 mL of a mixed solution of chloroform-methanol (4:1) was added, weighed, mixed well, heated, and refluxed in a water bath at 80 °C for 2 h, cooled, and weighed again. The above-mixed solution was added and filtered to make up for the lost weight. Then, 10 mL of the filtrate was precisely measured, evaporated in an evaporating dish, the residue dissolved with methanol, and transferred to a 2 mL volumetric flask. Methanol was then added to the scale, and the solution was mixed well to obtain the test solution. The peimine and peiminine were set as reference substances, and the signal peak area was determined by HPLC-ELSD. The content of peimine and peiminine in different treatments was calculated. According to the Chinese Pharmacopoeia, the total amount of peimine and peiminine should not be less than 0.08%.

### 2.4. Determination of Soil Factors

Total potassium (TK) was determined by sodium hydroxide melting method, available potassium (AK) was determined by sodium tetraphenylborate turbidimetry, soil pH was determined by pH meter, available phosphorus (AP) was determined using the molybdenum antimony anti-colorimetric method, alkali-hydrolyzed nitrogen (HN) was determined by Kjeldahl method [25]. Soil urease (S-UE) and soil acid phosphatase (S-ACP) were determined by Nanjing Jiancheng Kit (Qiagen, Nanjing, China).

### 2.5. Rhizosphere Microbial Community Analysis

A total of 0.2 g of the sample was weighed, and the microbial DNA was extracted by E.Z.N.A.^®^ soil DNA Kit (Omega Bio-tek, Norcross, GA, USA). The purity and concentration of the sample DNA were detected by NanoDrop2000 spectrophotometer (Thermo Scientific, MA, USA). The integrity of the extracted DNA was detected by 1% agarose gel electrophoresis. The specific primers with barcodes were synthesized according to the sample detection area for PCR amplification. Amplification of bacterial 16S rRNA gene in rhizosphere soil Two rounds of primers in the V3−V4 region were selected. The first round of primers 799F (5′-AACMGGATTAGATACCCKG-3′) and 1392R (5′-ACGGGCGGTGTGTGTRC-3′), the second round of primers 799F (5′-AACMGGATTAGATACCCKG-3′) and 1193R (5′-ACGTCATCCCCACCTTCC-3′); ITS amplification of rhizosphere soil fungi Primers ITS1F (5′-CTTGGTCATTTAGAGGAAGTAA-3′) and ITS2R (5′-GCTGCGTTCTTCATCGATGC-3′) were selected from the ITS1−ITS2 region, which were provided by Majorbio Bio-Pharm Technology Co., Ltd. (Majorbio Bio-Pharm Technology Co. Ltd., Shanghai, China).

### 2.6. Data Analysis

Bioinformatic analysis of the soil microbiota was carried out using the Majorbio Cloud platform (https://cloud.majorbio.com, accessed on 6 June 2024). The differences among the microbial communities in different samples were determined by principal coordinate analysis (PCoA) based on weighted unifrac distance using the Vegan v2.5-3 package. The linear discriminant analysis (LDA) effect size (LEfSe) (http://huttenhower.sph.harvard.edu/LEfSe, accessed on 8 August 2024) was performed to identify the significantly abundant taxa (genera) of bacteria among the different groups (LDA score > 3, *p* < 0.05). SPSS ver. 20.0 and GraphPad Prism 9.0 were employed for statistical analysis and mapping. One-way analysis of variance (ANOVA) was used to examine sample difference significance (*p* < 0.05).

## 3. Results

### 3.1. Effects of Shading, K Application, and Their Coupling Treatments on F. thunbergii Biomass

**Figure 2:**
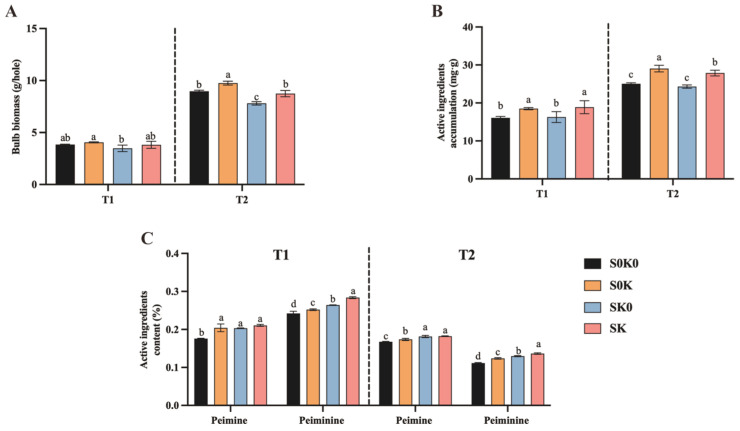
Effects of individual shading (SK0), individual K application (S0K), and their coupling (SK) treatments on the bulb of *F. thunbergii*. (**A**) Bulb biomass at the T1 and the T2. (**B**) Bulb active ingredient content accumulation at the T1 and the T2. (**C**) Bulb active ingredients content at the T1 and the T2. Lowercase letters indicate significant differences under different treatments (*p* < 0.05).

At the T1, compared with S0K0, SK0, S0K, and SK treatments, had no significant effect on bulb biomass (Figure 2A). At the T2, compared with S0K0, SK0 treatment significantly reduced bulb biomass by 13.0%. S0K treatment significantly increased bulb biomass by 8.8%, and SK treatment reduced biomass by 2.5% but did not reach a significant level. The bulb biomass of the SK treatment increased by 12.0% compared with that of the SK0 treatment. This indicates that K application after shading can make up for the loss of bulb biomass caused by shading.

### 3.2. Effects of Shading, K Application, and Their Coupling Treatments on the Content and Accumulation of Active Ingredients in F. thunbergii

At the T1 (Figure 2C), compared with those in S0K0, the peimine and peiminine contents in SK0, S0K, and SK treatments increased significantly, by 15.6%, 16.2% and 19.6%, 9.1%, 4.1%, and 17.1%, respectively. At the T2, compared with S0K0, the peimine and peiminine contents in SK0, S0K, and SK treatments increased significantly by 3.8%, 8.4%, and 8.6%, and 16.1%, 10.9%, and 22.0%, respectively. After SK0, S0K, and SK treatments, the peimine content in *F. thunbergii* increased significantly in the T1, the peimine content increased significantly in the T2, and the content of active ingredients in the coupling treatment was the highest.

According to the biomass and active ingredient content in the T1 (Figure 2B), compared with those in S0K0, the accumulation of active ingredients increased by 15.0%, 1.2%, and 17.4% after SK0, S0K, and SK treatments, respectively. In the T2, the accumulation of active ingredients increased by 15.9% after S0K treatment, decreased by 2.9% after SK0, and increased by 11.3% after SK treatment. Compared with that in the SK0, the active ingredient content of SK treatment in the T2 was significantly increased by 14.6%. The application of K after shading can increase the accumulation of active ingredients in bulbs of *F. thunbergii*, and the degree of influence on the accumulation of active ingredients varied at different growth stages.

### 3.3. Effects of Shading, K Application, and Their Coupling Treatments on Soil Factors

**Figure 3:**
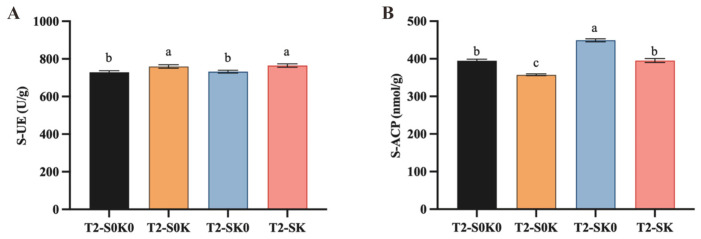
Effects of individual shading (SK0), individual K application (S0K), and their coupling (SK) treatments on soil factors. (**A**) Soil urease. (**B**) Soil acid phosphatase. Lowercase letters indicate significant differences under different treatments (*p* < 0.05).

As shown in Table 1 and Figure 3, shading significantly affected AP, pH, and S-ACP (*p* < 0.05). Compared with those in S0K0, AP and pH decreased by 0.9% and 8.4%, respectively, and S-ACP increased by 13.8% after SK0 treatment. S0K treatment significantly affected the AK, AN, pH, S-UE, and S-ACP (*p* < 0.05). Compared with the S0K0, S0K treatment increased AK and S-UE by 29.1% and 4.2%, respectively, and decreased AN and pH by 5.5% and 0.6%, respectively. The SK treatment significantly affected AK, AP, pH, and S-UE, with amplitudes of +21.9%, −26.4%, −6.9%, and +4.4%, respectively. K fertilizer affected more soil factors, and the changes in most soil factors in the coupling treatment were consistent with those of K application.

### 3.4. Microbial Diversity and Community Composition Responses to Combined Shading and K Application

#### 3.4.1. Alpha Diversity Index of Bacterial and Fungal Communities

To examine the changes in the microbial community in the rhizosphere of *F. thunbergii* under shading, K application, and coupling conditions, the bacterial and fungal genomes were sequenced using 16S rRNA gene sequencing under K application, shading, and coupled K application and shading. Different *F. thunbergii* rhizosphere microbial communities were formed by shading and K application. By optimizing the extraction, 3,224,023 high-quality clean bacterial reads passed the high-quality screening, and most of the sequence lengths were 351–400 bp. A total of 3,258,817 high-quality clean fungal reads passed the high-quality screening, and most sequence lengths were 201–250 bp. The OTU sequences with 97% similarity were clustered among various treatments. In total, 15,695 bacterial OTU effective sequences and 2426 fungal OTU effective sequences were obtained from the *F. thunbergii* rhizosphere soil. According to the sequencing quantity and depth of the samples, the dilution curves of bacteria and fungi gradually flattened. This indicated that the sequencing sample size met the requirements and was representative of the *F. thunbergii* rhizosphere microorganisms (Supplementary Tables S1 and S2, and Figure S1).

The richness and diversity indices of the microbial community are listed in the table (Supplementary Tables S3 and S4). The *p* value was calculated using Tukey’s test after a pairwise comparison of the corresponding values of each index. There were no significant differences in these indices.

#### 3.4.2. Beta Diversity Index of Bacterial and Fungal Communities

**Figure 4:**
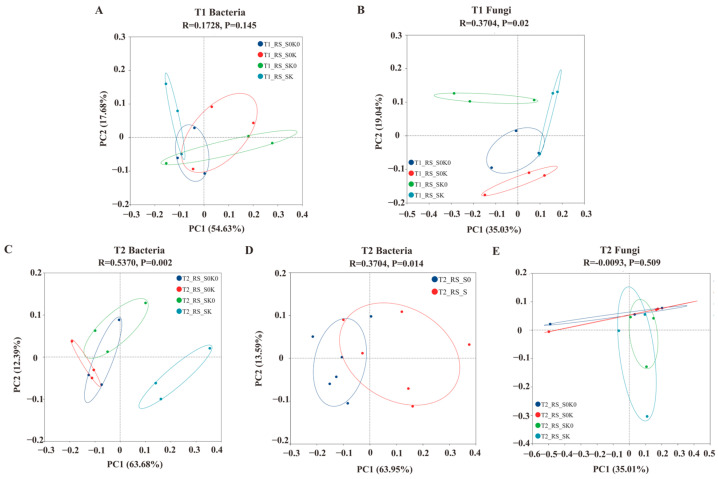
Principal coordinate analysis (PCoA) on bacterial and fungal community compositions in the rhizosphere soil of *F. thunbergii* under different treatments. (**A**) PCoA of bacteria at the T1. (**B**) PCoA of bacteria at the T2. (**C**) PCoA of bacteria at the T2, S vs. S0. (**D**) PCoA of fungi at the T1. (**E**) PCoA of fungi at the T2.

Principal component analysis (PCoA) was performed to observe the similarities and differences in the rhizosphere soil microbial communities between the different treatments. The bacterial community distances in the different treatments during the T1 were close together (Figure 4A). This indicated that the bacterial communities in the rhizosphere of the different treatments during this period were similar. The contribution rate of the first principal component (PC1) of the bacterial community to the sample difference in the T2 was 63.68%, and the contribution rate of the second principal component (PC2) was 12.39% (Figure 4C). In PC1, the bacterial community composition of SK was distinguished from that of S0K0, S0K, and SK0, indicating that the composition and structure of the bacterial community of the shading and K coupling treatment in the T2 were significantly different from those of the other treatments (*p* < 0.001). A comparison of the K application and shading treatments revealed that the bacterial community composition and structure of the shading and non-shading groups at the T2 were different and reached a significant level (Figure 4D). This indicated that the shading treatment had a greater impact on the bacterial community composition at the later stage. Different treatments had different effects on the fungal community composition and bacteria, mainly during the T1 (Figure 4B). The contribution rate of PC1 of the fungal community to the sample difference during the T1 was 35.03%, and the contribution rate of PC2 was 19.04%. The composition of the fungal community in the PC2, S0K0, S0K, SK0 was separated from each other. This indicated that the fungal community composition and structure were significantly different from those in the other treatments (*p* < 0.05) under the shading and K application treatments in the T1.

#### 3.4.3. Rhizosphere Microbial Community Structure

**Figure 5:**
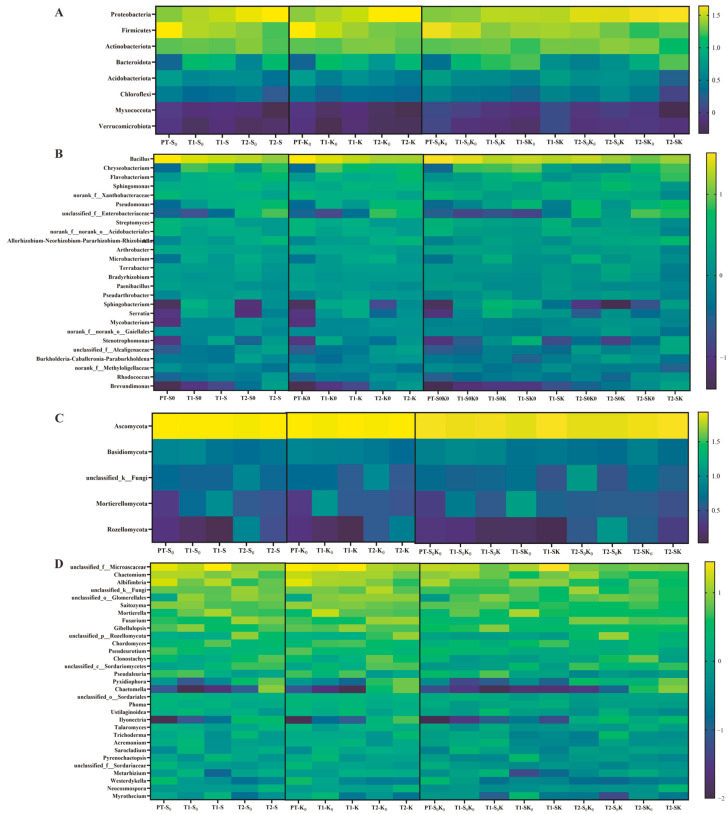
Effects of individual shading (SK0), individual K application (S0K), and their coupling (SK) treatments on the relative abundance of rhizosphere microorganisms. (**A**) Heat map of relative abundance of bacteria at the phylum level. (**B**) Heat map of relative abundance of bacteria at the genus level. (**C**) Heat map of relative abundance of fungi at the phylum level. (**D**) Heat map of relative abundance of fungi at the genus level.

To examine the microbial community structure from the samples, the abundance distribution at the phylum and genus levels was analyzed. At the phylum level, a total of 36 rhizosphere bacterial phyla were detected, and eight of them with relative abundance > 1% were *Proteobacteria*, *Firmicutes*, *Actinobacteriota*, *Bacteroidota*, *Acidobacteriota*, *Chloroflexi*, *Myxococcota*, and *Verrucomicrobiota* (Figure 5A). Among them, *Proteobacteria*, *Firmicutes*, *Actinobacteriota*, and *Bacteroidota* were the main four bacterial phyla in all treatment groups, accounting for approximately 80% of all bacteria. Compared with that in S0K0, the relative abundance of *Proteobacteria* increased, and the relative abundance of *Firmicutes* decreased in the SK0, S0K, and SK treatments, especially during the T2. The relative abundance of *Actinobacteriota* in SK0, S0K, and SK treatments was less affected at the T1 and decreased at the T2. The relative abundance of *Bacteroidota* in the SK0 and S0K treatments decreased in the T1 and increased in the T2. The relative abundance of *Bacteroidota* in the SK treatment increased in both periods.

**Figure 6:**
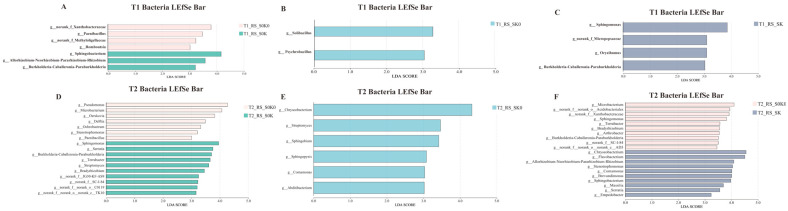
Linear discriminant analysis (LDA) based on the level of bacterial microbial genera showed the rhizosphere microbial classes that caused significant differences between different treatments at the T1 and the T2, LDA > 3, *p* < 0.05. S0K0 vs. S0K (**A**,**D**); S0K0 vs. SK0 (**B**,**E**); S0K0 vs. SK (**C**,**F**).

At the genus level, 1091 rhizosphere soil bacterial genera were identified. As shown in Figure 5B, *Bacillus*, *Chryseobacterium*, *Flavobacterium*, *Sphingomonas*, and *Pseudomonas* were mainly present in the rhizosphere soil of *F. thunbergii* (Figure 5 and Figure 6). Linear discriminant analysis (LDA) effect size (LEfSe) was employed to compare microbial communities and identify specific microorganisms of *F. thunbergii* rhizosphere responding to SK0, S0K, and SK treatments. The bacterial genera that were significantly enriched in SK0 include *Chryseobacterium* and *Streptomyces*. In S0K, the bacterial genera that were significantly enriched are *Sphingobacterium*, *Allorhizobium-Neorhizobium-Pararhizobium-Rhizobium* (ANPR), *Burkholderia-Caballeronia-Paraburkholderia* (BCP), *Sphingomonas*, *Serratia*, *Terrabacter*, *Streptomyces*, and *Bradyrhizobium*. The bacterial genera significantly enriched in SK are *Chryseobacterium*, *Sphingobacterium*, ANPR, BCP, *Sphingomonas*, *Brevundimonas*, and *Spingobacterium*. Additionally, the relative abundance of bacterial genera not only responded to the treatments but also to the changes in different growth stages. Those with gradually increasing relative abundance over the growth stages include *Pseudomonas*, *Allorhizobium-Neorhizobium-Pararhizobium-Rhizobium* (ANPR), *Microbacterium*, and *Sphingobacterium*, whereas *Streptomyces* showed a gradual decrease in relative abundance over the growth stages.

At the phylum level of fungal community structure, a total of 15 fungal phyla were detected, with 5 having a relative abundance greater than 1%. These are *Ascomycota*, *Basidiomycota*, *unclassified_ k_ Fungi*, *Rozellomycota*, and *Mortierellomycota*, which together account for approximately 90% of all fungi (Figure 5C). Relative to the S0K, the relative abundance of *Ascomycota* saw an increase in the T2 under SK0, S0K, and SK treatments. For *Basidiomycota*, a decline in relative abundance was observed in the T1 with shading and in the T2 with K application. Additionally, a reduction was noted across both periods under the combined effects of shading and K application. The relative abundance of *unclassified_ k_ Fungi* exhibited a decrease in both periods when subjected to SK0, S0K, and SK treatments. The relative abundance of *Mortierellomycota* rose in the T1 with shading but fell in the T2. It also showed a decrease in both periods under K application, mirroring the trend observed under shading when both factors were applied together. Lastly, the relative abundance of *Rozellomycota* dropped in the T1 with both shading and K application; it rebounded in the T2, while under the combined factors, it experienced a decline across both periods.

**Figure 7:**
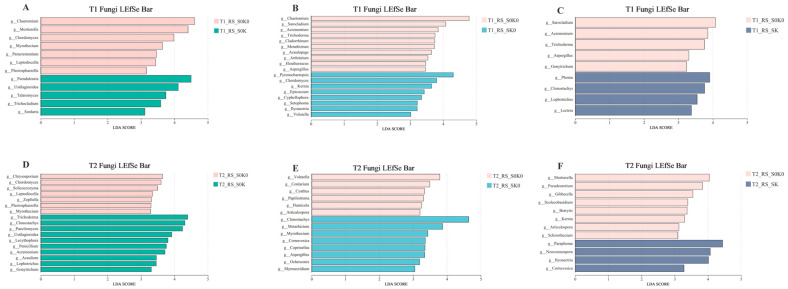
Linear discriminant analysis (LDA) based on the level of fungal microbial genera showed the rhizosphere microbial classes that caused significant differences between different treatments at the T1 and the T2, LDA > 3, *p* < 0.05. S0K0 vs. S0K (**A**,**D**); S0K0 vs. SK0 (**B**,**E**); S0K0 vs. SK (**C**,**F**).

A total of 442 rhizosphere soil fungal genera were detected at the genus level. Unclassified_ f_ *Microascaceae*, *Chaetomium*, *Albifimbria*, unclassified_ k_ Fungi, unclassified_ o_ *Glomerellales*, *Saitozyma*, *Mortierella*, and *Fusarium* are found mainly in the rhizosphere soil (Figure 5 and Figure 7). The fungal genera that were significantly enriched in SK0 include *Pyrenochaetopsis*, *Chordomyces*, *Ilyonectria*, *Clonostachys*, *Metarhizium*, and *Myrothecium*. The fungal genera enriched in S0K are *Pseudaleuria*, *Trichoderma*, *Ustilaginoidea*, and *Talaromyces*. The fungal genera significantly enriched in SK are *Phoma*, *Clonostachy*, *Neocosmospera*, and *Ilyonectria*. The relative abundance of dominant fungal genera in the rhizosphere of *F. thunbergii* varies with the growth and development period. The genera whose relative abundance increases over the growth stages include *Ilyonectria*; those whose relative abundance decreases over the growth stages include *Albifimbria*, *Pseudeurotium*, and *Pseudaleuria*, among others.

### 3.5. Correlation Analysis

#### 3.5.1. Relationships Between Soil Factors and Biomass and Active Ingredient Content of *F. thunbergii* in Shade, K Application, and Their Coupling Treatments

**Figure 8:**
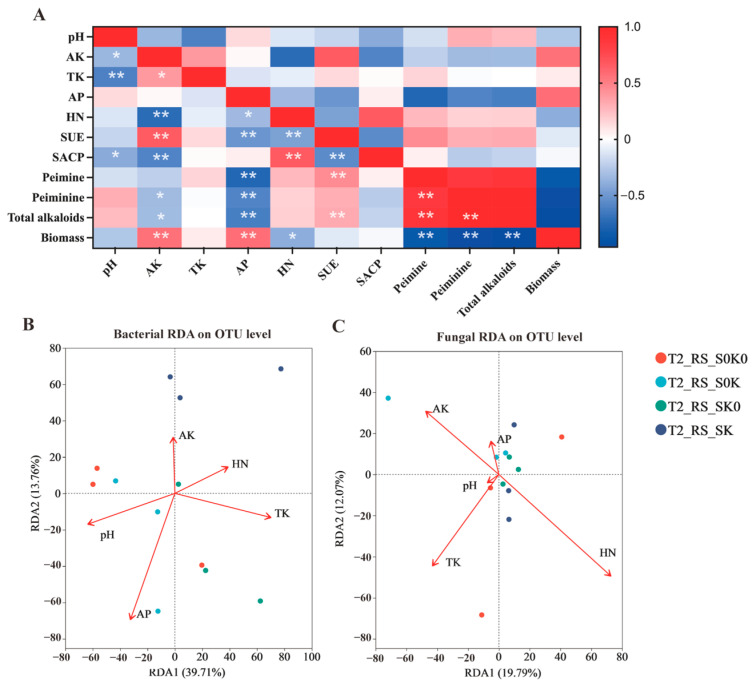
Heat map of correlation between soil factors and alkaloid biomass (**A**), RDA analysis between soil factors and bacterial communities (**B**), RDA correlation between soil factors and fungal communities (**C**). pH, Soil pH; AK, Available potassium; TK, Total potassium; HN, Alkali-hydrolyzed nitrogen; AP, Available phosphorus; S-UE, Soil urease; S-ACP, Soil acid phosphatase. The asterisk (*) indicates a statistically significant difference. (* *p* < 0.05; ** *p* < 0.01).

The correlation analysis heat map (Figure 8A) showed that the bulb biomass of *F. thunbergii* was significantly positively correlated with the soil factors AK and AP and significantly negatively correlated with HN. The content of the active ingredients in *F. thunbergii* was significantly negatively correlated with the soil factors AK and AP. The bulb biomass of *F. thunbergii* was significantly and negatively correlated with its active ingredients.

#### 3.5.2. Relationship Between Rhizosphere Microorganisms and Biomass and Active Ingredient Content of *F. thunbergii* in Shade, K Application, and Their Coupling Treatments

**Figure 9:**
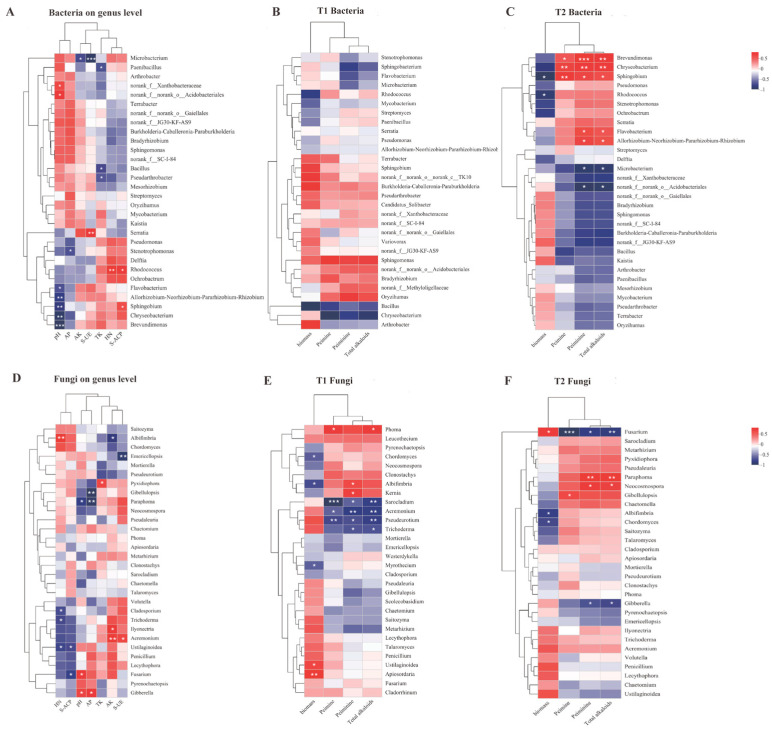
The relationship between soil factors, bulb biomass, bulb alkaloids, and rhizosphere microorganisms. Correlation heatmap of soil factors with bacterial genera (**A**) and fungal genera (**D**). Correlation heatmap of bulb biomass and active ingredients content with bacterial genera (**B**) and fungal genera (**E**) at the T1. Correlation heatmap of bulb biomass and active ingredients content with bacterial genera (**C**) and fungal genera (**F**) at the T2. pH, Soil pH; AK, Available potassium; TK, Total potassium; HN, Alkali-hydrolyzed nitrogen; AP, Available phosphorus; S-UE, Soil urease; S-ACP, Soil acid phosphatase. The asterisk (*) indicates a statistically significant difference according to Spearman Correlation (* *p* < 0.05; ** *p* < 0.01 and *** *p* < 0.001).

No bacterial genera were significantly related to biomass or active ingredient content during the T1 (Figure 9B,C). During the T2, *Sphingobium* and *Rhodococcus* were significantly negatively correlated with biomass. During the T2, the genera that were significantly positively correlated with the content of active ingredient content were *Sphingobium*, *Chryseobacterium*, *Brevundimonas*, *Flavobacterium*, and ANPR. *Microbacterium* and *norank_ f_ norank_ o_ Acidobacteriales* were significantly negatively correlated with peiminine. The number of bacterial genera significantly associated with peiminine in the T2 was more than that of peiminine.

During the T1 (Figure 9E,F), *Ustilaginoidea* and *Apiosordaria* were significantly positively correlated with biomass. Meanwhile, *Myrothecium*, *Chordomyces*, and *Albifimbria* were significantly negatively correlated with biomass. During the T1, the genera *Albifimbria*, *Kernia*, and *Phoma* were significantly positively correlated with the content of active ingredient content, and *Trichoderma*, *Acremonium*, *Pseudeurotium*, and *Sarocladium* were significantly negatively correlated with the content of active ingredient content. During the T2, *Fusarium* was significantly positively correlated with biomass, and *Albifimbria* and *Chordomyces* were significantly negatively correlated with biomass. During the T2, *Paraphoma*, *Neocosmospora*, and *Gibellulopsis* were significantly positively correlated with the content of active ingredients, and *Fusarium* and *Gibberella* were significantly negatively correlated.

#### 3.5.3. Relationship Between Rhizosphere Microorganisms and Soil Factors of *F. thunbergii* Under Shading and K Application

According to the redundancy analysis (RDA), pH and AP significantly affected the bacterial community, whereas AK and HN significantly affected the fungal community (Figure 8B,C).

Among the bacteria (Figure 9A), *Flavobacterium*, ANPR, *Sphingobium*, *Chryseobacterium*, and *Brevundimonas* were significantly negatively correlated with pH, and *norank_ f_ Xanthobacteraceae* and *norank_ f_ norank_ o_ Acidobacteriales* were significantly positively correlated with pH. *Stenotrophomonas* was significantly negatively correlated with AP. *Microbacterium* was significantly negatively correlated with AK. *Bacillus*, *Pseudarthrobacter*, and *Paenibacillus* were significantly negatively correlated with TK. *Rhodococcus* was significantly positively correlated with HN. *Serratia* was significantly positively correlated with S-UE, and *Microbacterium* was significantly negatively correlated with S-UE. *Rhodococcus* and *Sphingobium* were significantly positively correlated with S-ACP.

Among the fungi (Figure 9D), *Fusarium* and *Gibberella* were significantly positively correlated with pH, and *Paraphoma* was significantly negatively correlated with pH. *Gibberella* was significantly positively correlated with AP, and *Paraphoma* and *Gibellulopsis* were significantly negatively correlated with AP. *Ilyonectria* and *Acremonium* were significantly positively correlated with AK, and *Albifimbria* was significantly negatively correlated with AK. *Pyxidiophora* was significantly positively correlated with TK. *Albifimbria* was significantly positively correlated with HN, and *Cladosporium*, *Trichoderma*, and *Ustilaginoidea* were significantly negatively correlated with HN. *Acremonium* was significantly positively correlated with S-UE, while *Emericellopsis* was significantly negatively correlated. *Fusarium* and *Ustilaginoidea* were significantly negatively correlated with S-ACP.

## 4. Discussion

### 4.1. Coupling Shading and K Application Can Achieve Yield Stability and Quality Improvement of F. thunbergii in a Certain Range

In this study, SK0 treatment significantly increased the content of active ingredients in *F. thunbergii*. The total amount of peimine and peiminine in the T1 increased by 11.9%, and the total of peimine and peiminine in the T2 increased by 11.5% (Figure 2C). Under light stress in other medicinal plants, shading increased the content of active ingredients in medicinal plants. The total saponins in the 40% shading group increased by 1.36% compared with the non-shading group. 50% shading significantly increased the 5,7-dimethoxyflavone of *Kaempferia parviflora* by 8.8% [26,27]. Shading increased the contents of different types of medicinal components to different degrees. This may be related to the synthetic metabolic pathways of different medicinal components affected by shading. Shading reduces the quality of crops. Shading changes the starch structure of rice and increases its chalkiness, thereby reducing its quality [28]. This is because the quality of crops is mainly determined by primary metabolites, such as starch, protein, and fat. Starch is the main storage material in crops and one of the main factors affecting crop yield. Therefore, shading reduces crop quality and yield. SK0 treatment reduced the bulb biomass of *F. thunbergii*, which decreased by 9.5% in the T1 and significantly decreased by 13.0% in the T2 (Figure 2A). Both short-term and long-term shading had negative effects on the biomass of *F. thunbergii*.

The application of K fertilizer is beneficial for the synthesis, transportation, and storage of crop carbohydrates, and it has a positive effect on improving quality [14]. Previous studies in our laboratory have shown that K fertilizer can synergistically improve the biomass accumulation and quality of *F. thunbergii* [17]. Therefore, in the study, we attempted to use K treatment to compensate for the loss of biomass caused by shading and to examine whether combined shading and K application can achieve a high-quality and stable yield. The total alkaloids were significantly increased by 9.2% and 6.6% at the T1 and the T2, respectively (Figure 2C). This confirmed that the increase in K fertilizer played a key role in the increasing *F. thunbergii* biomass and active ingredient content.

SK treatment increased significantly the content of the active ingredients in *F. thunbergii* by 18.1% and 14.0% during the T1 and T2, respectively (Figure 2C). K application and shading significantly increased the effective component content of *F. thunbergii*, respectively. The effective component content of shading and K application coupled was also significantly higher than that of a single factor. This indicated that shading and K application have a synergistic effect on the improvement in the effective component content. Fertilization measures have a positive effect on plant biomass and yield under abiotic stress [29,30]. The SK treatment had a positive effect on biomass, whereas there was little change under the non-shading treatment, which was 9.0% and 12.0% higher than that under the shading condition (Figure 2A). This indicated that K application could partially be compensated for the biomass reduction caused by shading. We calculated the accumulation of medicinal components as the bulb biomass multiplied by the corresponding alkaloid content (Figure 2B). The accumulation of active ingredients in the shading and K application coupling treatment was the highest, indicating that coupled shading and K application significantly increased the accumulation of active ingredients in *F. thunbergii*. In summary, the coupling treatment could achieve stable yield and quality improvement of *F. thunbergii* within a certain range.

### 4.2. Effects of Shading, K Application, and Their Coupling Treatments on Soil

K application and coupling factors in soil increased AK significantly (Figure 3 and Table 1). S-UE activity increased significantly, and all the treatments reduced pH. K^+^ could directly regulate the soil nitrogen cycle enzyme activity [31]. AK and S-UE activities were significantly increased after K fertilization, which could improve the soil nitrogen conversion intensity and regulate the absorption of soil nitrogen by *F. thunbergii*. This could significantly increase the biomass of bulbs of *F. thunbergii*. Alkaloids are a class of nitrogen-containing organic compounds, and nitrogen absorbed by the soil may promote the synthesis of alkaloids in bulbs [32]. K application reduces soil pH, which may be because plant roots release H^+^ to the soil while absorbing cations, thereby reducing pH [33]. Therefore, shading, K application, and coupled treatments significantly affected soil factors. In turn, this affects the growth and development of medicinal plants and the synthesis of active ingredients.

### 4.3. Shading, K Application, and Their Coupling Treatments Changed the Rhizosphere Microbial Structure

*Proteobacteria*, *Firmicutes*, *Actinobacteriota*, and *Bacteroidota* were the dominant bacterial phyla in the rhizosphere soil of *F. thunbergii*. *Ascomycota*, *Basidiomycota*, *Rozellomycota*, and *Mortierellomycota* were the dominant fungal phyla. This is consistent with the dominant phyla of rhizosphere microorganisms in American ginseng [34] and yam [35]. The universality of these phyla may be related to the diverse metabolic capabilities of many members at the gate level and their ability to decompose organic matter [36]. Shading, K application, and coupling treatments mainly enriched these categories at the phyla level.

Shading, K application, and coupling factors significantly altered the community structure of rhizosphere microorganisms at the genus level (Figure 5, Figure 6 and Figure 7). For bacteria, *Chryseobacterium*, *Sphingobacterium*, ANPR, BCP, *Sphingomonas*, *Brevundimonas*, and *Spingobacterium* were enriched under the coupling factor of shading and K application. *Streptomyces* were enriched in both shading and K application treatments. *Proteobacteria* and *Actinobacteriota* were the most abundant bacteria in the rhizosphere under shading and coupling treatments. These two phyla respond to various environmental stresses, and *Actinobacteriota* was involved in promoting plant growth under stress. Many of these phyla form spores that are resistant to adverse conditions and can survive under stressful conditions [37,38,39,40,41]. Among the dominant bacteria responding to each treatment, *Brevundimonas* responded to coupling treatment, BCP responded to K application and coupling treatments, and *Streptomyces* responded to K application and shading treatments, all of which could improve nitrogen use efficiency and promote plant growth and development [42,43,44]. In response to K application and coupling treatments, *Sphingomonas* converts N, P, K, organic matter, and trace elements into nutrients that can be absorbed by crops, thereby promoting plant growth [45].

Among fungi, *Phoma*, *Cloonostachy*, *Neocosmospera*, and *Ilyonectria* were enriched under coupling treatment. *Pyrenochaetopsis*, *Chordomyces*, *Ilyonectria*, *Clonostachys*, *Metarhizium*, and *Myrothecium* were enriched in shading treatment. *Pseudaleuria*, *Trichoderma*, *Ustilaginoidea*, and *Talaromyces* were enriched in the K application treatment. Notably, *Trichoderma* responded to K application treatment according to the dominant fungus enriched in each treatment. *Trichoderma* significantly enhanced plant height, weight, and yield during yam cultivation [35]. *Trichoderma* may be a beneficial fungus that promotes the growth and development of *F. thunbergii* after K application.

Changes in rhizosphere microbial communities are closely related to plant growth stages and environmental conditions, and changes in these microbial communities have a substantial impact on plant growth and development [46]. The relative abundance of microorganisms in the rhizosphere of *F. thunbergii* changed with the growth period. For bacteria, ANPR, BCP, *Brevundimonas*, and *unclassified_ f_ Enterobacteriaceae* had significant changes in their relative abundance. For fungi, the relative abundance of fungi changed significantly, such as *Chaetomium*, *Ilyonectria*, and *Myrothecium*. The relative abundance of *Ilyonectria* increased gradually with time. Studies on the medicinal plant *Alkanna tinctoria* have shown that the relative abundance of ANPR is affected by the developmental stage and is significantly related to secondary metabolites. It is a core rhizosphere microorganism that promotes the synthesis of medicinal components at different developmental stages of medicinal plants [47]. The abundance of ANPR increased with the growth stages of *F. thunbergii*, indicating that ANPR may be a beneficial bacterium that promotes the synthesis of effective components of *F. thunbergii* in response to growth stages.

### 4.4. Relationship Between Soil Factors and Key Microorganisms and the Growth, Development, and Quality of F. thunbergii

Soil AK was significantly and positively correlated with the bulb biomass of *F. thunbergii* (Figure 8A). Notably, soil AK in the soil increases the cytoplasmic concentration in roots and enhances the absorption of water and nutrients by plant roots, thus promoting material accumulation in plants [14]. The RDA results showed that pH and AP significantly affected the bacterial community. AK and HN significantly affected fungal communities. The available K content in soil affects the structure and diversity of the microbial community [48]. pH is the soil factor most significantly related to microorganisms (Figure 8B,C), and is the decisive factor affecting bacterial diversity and community composition [49].

The medicinal part of *F. thunbergii* is the underground bulb. Changes in rhizosphere microorganisms play an important role in its growth and active ingredient synthesis of *F. thunbergii.* The correlation heat map (Figure 9) showed that the bacteria genera were significantly related to biomass and alkaloids in the T2. These effects were mainly concentrated in the significant correlation with the active ingredients. The number of bacteria with a significant positive correlation with the active ingredient content was ANPR, *Brevundimonas*, *Flavobacterium*, *Chryseobacterium*, etc. Among them, ANPR and *Brevundimonas*, which have nitrogen fixation, have significant impacts on nutrient transformation, thereby promoting plant growth [44]. Some nitrogen-fixing bacteria can induce the MEP pathway gene expression and affect the synthesis of active ingredients [50]. The metabolic pathways of active ingredients in *F. thunbergii* are the mevalonate pathway (MVA) and the methylerythritol 4-phosphate pathway (MEP). These bacteria may also affect the synthesis of peiminine by altering gene expression in the alkaloid synthesis pathway. The relative abundances of ANPR and *Brevundimonas* increased under the coupling treatment of shading and K application. Therefore, they may be the key bacteria that promote the synthesis of effective components of *F. thunbergii* in rhizosphere soil after shading and K application. Moreover, *Chryseobacterium* was significantly positively correlated with the active ingredients, and it was also positively correlated with biomass at the T1. This can produce indole acetic acid (IAA) to promote plant growth [51]. *Chryseobacterium* may be involved in the coordination of the growth and development of *F. thunbergii* bulbs at all stages.

*Albifimbria* and *Phoma* were significantly and positively correlated with alkaloids during the T1. Among them, *Phoma* can synthesize the terpenoids *Phomadecalin* C [52]. The synthesis pathway of terpenoids is the same as that of the alkaloid precursors. The relative abundance of *Phoma* also increased after shading and K application coupling treatment. This fungus may be the key fungus that promotes alkaloid synthesis. The significantly enriched *Trichoderma* in K application treatment was positively correlated with biomass. *Trichoderma* can promote the growth and development of various crops and increase yield [53], indicating that the fungus may promote the growth and development of *F. thunbergii.*

We found that rhizosphere bacteria and fungi played an important role in the growth, alkaloid synthesis, and accumulation of *F. thunbergii* in response to shading, K application, and their combination. However, relatively more bacteria were involved in regulating the growth and development of *F. thunbergii* and the synthesis of active ingredients.

## 5. Conclusions

The results of this research indicate that shading led to an 11.5–11.9% increase in the content of active ingredients in bulbs while causing a decrease in biomass by 9.5–13.0%, resulting in a reduced accumulation of active ingredients. The combination of shading and potassium application alleviated the reduction in biomass induced by shading while facilitating the accumulation of effective components in *F. thunbergii*. The coupling factor was enriched in *Chryseobacterium*, *Sphingobacterium*, ANPR, BCP, *Sphingomonas*, *Brevundimonas*, and *Phoma*. *Chryseobacterium*, *Brevundimonas*, and *Phoma* exhibited significant positive correlations with medicinal components. BCP and *Brevundimonas* also responded to changes at different growth stages of *F. thunbergii*, indicating that these microorganisms were beneficial for promoting its growth and synthesis of active ingredients after shading and K application in the rhizosphere microorganisms of *F. thunbergii*. Furthermore, Shading, K application, and coupling factors reduced pH, whereas K application and coupling factors increased AK and S-UE. These factors were closely related to the changes in the rhizosphere microbial community composition. The coupling treatment of shading and potassium application constitutes a feasible strategy for the cultivation of *F. thunbergii* with high quality and stable yield, within which rhizosphere microorganisms play a crucial role. The study illuminates the intricate relationships between bulb growth, soil properties, and rhizosphere microbial dynamics, providing effective reference and technical support for the cultivation regulation and soil improvement of rhizome medicinal plants.

## Figures and Tables

**Table 1:** Effects of individual shading (SK0), individual K application (S0K), and their coupling (SK) treatments on soil factors.

Treatment	AK mg/kg	TK g/kg	HN mg/kg	AP mg/kg	pH
PT-S0K0	235.50 ± 2.29 d	5.75 ± 0.10 a	205.33 ± 2.14 a	21.05 ± 1.02 c	5.49 ± 0.03 a
T2-S0K0	254.80 ± 7.81 c	5.64 ± 0.36 a	204.40 ± 3.70 a	22.36 ± 0.37 b	5.44 ± 0.29 a
T2-S0K	328.83 ± 5.11 a	5.85 ± 0.08 a	193.20 ± 2.42 b	22.64 ± 0.65 b	5.14 ± 0.02 b
T2-SK0	248.00 ± 3.77 c	5.82 ± 0.06 a	206.27 ± 3.52 a	20.77 ± 0.16 c	4.93 ± 0.04 bc
T2-SK	310.67 ± 3.79 b	5.87 ± 0.12 a	203.93 ± 2.14 a	24.69 ± 0.64 a	4.87 ± 0.05 c

AK, Available potassium; TK, Total potassium; HN, Alkali-hydrolyzed nitrogen; AP, Available phosphorus; pH, Soil pH. Lowercase letters indicate significant differences under different treatments (*p* < 0.05).

## Data Availability

The unprocessed information acquired from the sequencing procedure has been kept in the Sequence Read Archive (SRA) at the National Center for Biotechnology Information (NCBI) repository, identifiable by the accession number PRJNA1189600 and PRJNA1189607.

## Supplementary Materials

The following supporting information can be downloaded at: https://www.mdpi.com/article/10.3390/microorganisms13010125/s1, Figure S1: Dilution Curves and Rank-Abundance Curves of Rhizosphere Bacteria (A,B) and Rhizosphere Fungi (C,D) of *F. thunbergii*.; Table S1: Bacterial sequencing information; Table S2: Fungal sequencing information; Table S3: Bacterial alpha-diversity index; Table S4: Fungal alpha-diversity index.
