# Supplementary material for: The Combination of Shading and Potassium Application Regulated the Bulb Active Ingredients Accumulation in Fritillaria thunbergii Miq. by Affecting Rhizosphere Microecology
Source: Microorganisms. 2025 Jan 9;13(1):125. doi: 10.3390/microorganisms13010125 (PMC11767283; doi:10.3390/microorganisms13010125)
Supplement: Supplementary file 1 [file microorganisms-13-00125-s001.zip › 3 supplementary materials-20241204.pdf]

Table S1. Bacterial sequencing information

Treatment	Seq_num	Base_num	Mean_length	Min_length	Max_length
**PT-S_0K_0-1**	125363	47308701	377.37	208	429
**PT-S_0K_0-2**	113750	42913968	377.27	205	431
**PT-S_0K_0-3**	120317	45379040	377.16	203	428
**T1-S_0K_0-1**	120733	45469494	376.61	204	423
**T1-S_0K_0-2**	110090	41434786	376.37	203	425
**T1-S_0K_0-3**	121850	45976815	377.32	208	442
**T1-S_0K-1**	118814	44708275	376.29	203	410
**T1-S_0K-2**	124432	46879898	376.75	208	429
**T1-S_0K-3**	117239	44169146	376.74	212	414
**T1-SK_0-1**	123156	46290001	375.86	216	427
**T1-SK_0-2**	113066	42660145	377.30	205	419
**T1-SK_0-3**	117247	44096893	376.10	209	443
**T1-SK-1**	131064	49419019	377.06	211	415
**T1-SK-2**	114087	43024930	377.12	208	422
**T1-SK-3**	127973	48277984	377.25	205	431
**T2-S_0K_0-1**	120165	45309879	377.06	204	427
**T2-S_0K_0-2**	120830	45577154	377.20	205	439
**T2-S_0K_0-3**	110888	41810875	377.06	205	416
**T2-S_0K-1**	116174	43764221	376.71	204	425
**T2-S_0K-2**	118282	44581565	376.91	207	429
**T2-S_0K-3**	123042	46386374	377.00	212	425
**T2-SK_0-1**	112050	42199250	376.61	204	432
**T2-SK_0-2**	113732	42850592	376.77	214	430

**T2-SK$_0$-3**	126738	47759664	376.84	208	442
**T2-SK-1**	130160	48915848	375.81	208	401
**T2-SK-2**	123792	46572039	376.21	206	415
**T2-SK-3**	108989	41018065	376.35	202	419

Table S2. Fungal sequencing information

Treatment	Seq_num	Base_num	Mean_length	Min_length	Max_length
**PT-S_0K_0-1**	108832	25662359	235.797918	143	423
**PT-S_0K_0-2**	118760	28119035	236.77	140	425
**PT-S_0K_0-3**	118712	28229751	237.80	141	428
**T1-S_0K_0-1**	110703	25956718	234.47	140	430
**T1-S_0K_0-2**	113335	26333688	232.35	141	438
**T1-S_0K_0-3**	119366	28237404	236.56	140	438
**T1-S_0K-1**	107822	25742569	238.75	140	423
**T1-S_0K-2**	122467	28498873	232.71	142	426
**T1-S_0K-3**	126635	29858693	235.79	140	428
**T1-SK_0-1**	108140	24962096	230.83	140	430
**T1-SK_0-2**	111892	26588010	237.62	140	438
**T1-SK_0-3**	112624	26209639	232.72	140	438
**T1-SK-1**	118105	27541241	233.19	141	424
**T1-SK-2**	117179	27410046	233.92	141	425
**T1-SK-3**	116357	27223435	233.96	141	428
**T2-S_0K_0-1**	103968	24323231	233.95	140	429
**T2-S_0K_0-2**	122470	28791687	235.09	141	438
**T2-S_0K_0-3**	180001	45012464	250.07	140	438
**T2-S_0K-1**	129746	30154956	232.42	140	424
**T2-S_0K-2**	126288	29793792	235.92	141	426
**T2-S_0K-3**	120121	29314619	244.04	140	428
**T2-SK_0-1**	115030	27182076	236.30	141	429
**T2-SK_0-2**	130094	29730084	228.53	140	437

**T2-SK$_0$-3**	128295	30001269	233.85	140	438
**T2-SK-1**	123671	29223377	236.30	140	424
**T2-SK-2**	140126	30864058	220.26	142	426
**T2-SK-3**	108078	25662567	237.44	140	428

Table S3 Bacterial alpha-diversity index

Treatment	sobs	shannon	ace	chao	coverage
**PT-S_0K_0**	3401.33 ± 297.85	5.42 ± 0.41	4666.68 ± 367.28	4410.45 ± 320.42	0.98 ± 0.0017
**T1-S_0K_0**	3330.67 ± 242.92	5.47 ± 0.35	4692.16 ± 267.18	4390.46 ± 231.40	0.98 ± 0.0012
**T1-S_0K**	3020.00 ± 107.72	5.37 ± 0.33	4306.87 ± 135.65	4089.16 ± 166.57	0.98 ± 0.0006
**T1-SK_0**	3033.33 ± 353.16	5.13 ± 0.24	4403.30 ± 405.43	4102.76 ± 389.47	0.98 ± 0.0018
**T1-SK**	3675.67 ± 255.66	6.05 ± 0.38	5103.52 ± 297.02	4786.92 ± 283.31	0.97 ± 0.0013
**T2-S_0K_0**	3203.00 ± 203.70	5.64 ± 0.17	4568.97 ± 350.62	4239.97 ± 318.70	0.98 ± 0.0019
**T2-S_0K**	3191.67 ± 47.50	5.93 ± 0.16	4589.08 ± 35.72	4338.08 ± 60.54	0.98 ± 0.0002
**T2-SK_0**	3212.33 ± 264.63	5.78 ± 0.46	4534.93 ± 286.61	4287.80 ± 348.33	0.98 ± 0.0012
**T2-SK**	2815.33 ± 261.84	5.19 ± 0.22	4101.27 ± 319.12	3795.18 ± 287.46	0.98 ± 0.0015

Table S4 Fungal alpha-diversity index

Treatment	sobs	shannon	ace	chao	coverage
**PT-S_0K_0**	452.67 ± 32.13	3.81 ± 0.12	849.96 ± 54.54	681.06 ± 53.58	0.98 ± 0.0013
**T1-S_0K_0**	492.33 ± 38.89	4.00 ± 0.24	1019.79 ± 42.25	749.91 ± 25.87	0.98 ± 0.0003
**T1-S_0K**	485.00 ± 83.29	4.05 ± 0.31	852.06 ± 205.09	727.91 ± 120.99	0.98 ± 0.0033
**T1-SK_0**	496.33 ± 81.09	3.91 ± 0.14	840.24 ± 240.19	701.27 ± 154.84	0.98 ± 0.0041
**T1-SK**	427.00 ± 32.14	3.47 ± 0.29	857.02 ± 90.49	684.14 ± 53.45	0.98 ± 0.0014
**T2-S_0K_0**	455.00 ± 73.67	3.89 ± 0.36	834.03 ± 91.37	701.32 ± 85.23	0.98 ± 0.0016
**T2-S_0K**	462.00 ± 17.52	3.99 ± 0.32	800.50 ± 149.55	680.56 ± 58.41	0.98 ± 0.0017
**T2-SK_0**	483.00 ± 39.89	4.12 ± 0.22	958.28 ± 259.43	763.39 ± 147.05	0.98 ± 0.0031
**T2-SK**	474.33 ± 85.23	3.96 ± 0.35	1024.78 ± 275.87	776.21 ± 169.53	0.98 ± 0.0043

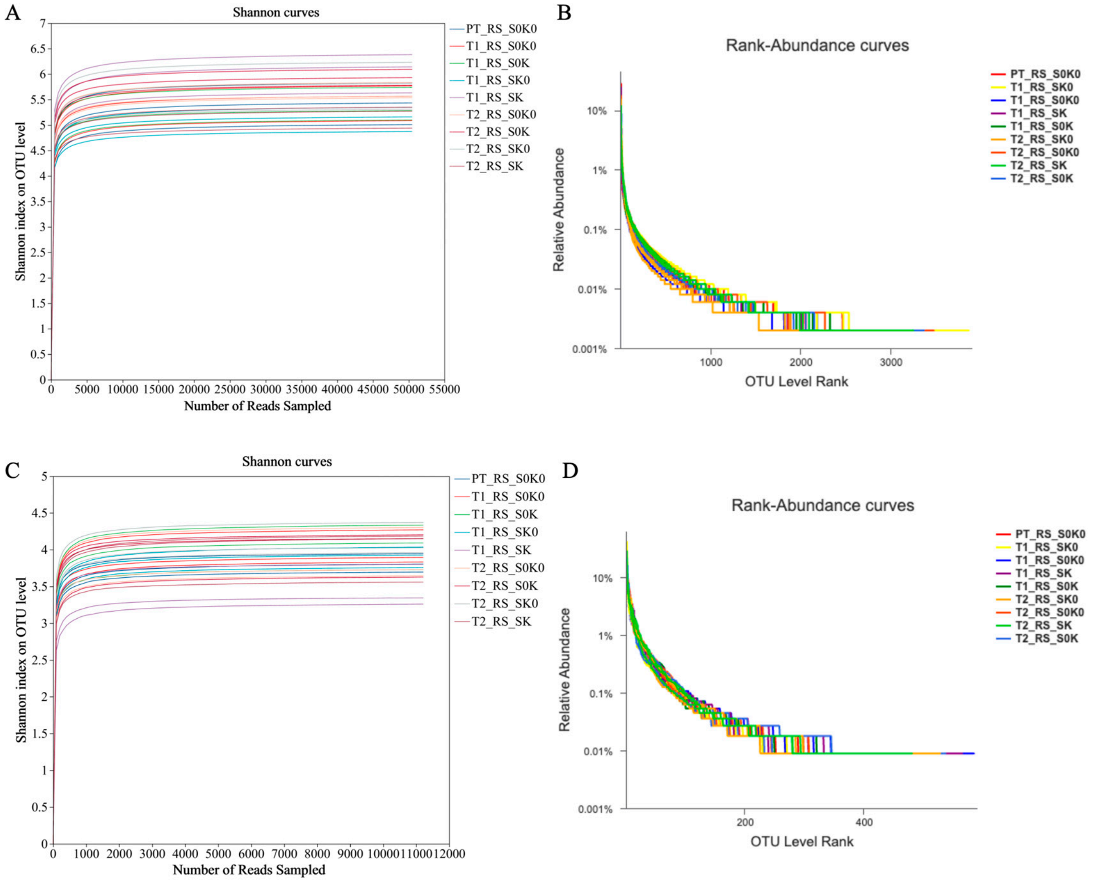

Figure S1 Dilution Curves and Rank-Abundance Curves of Rhizosphere Bacteria (A, B) and Rhizosphere Fungi (C, D) of *F.thunbergii.*
